# st‐DenseViT: A Weakly Supervised Spatiotemporal Vision Transformer for Dense Prediction of Dynamic Brain Networks

**DOI:** 10.1002/hbm.70364

**Published:** 2025-09-27

**Authors:** Behnam Kazemivash, Pranav Suresh, Dong Hye Ye, Armin Iraji, Jingyu Liu, Sergey Plis, Peter Kochunov, David C. Zhu, Vince D. Calhoun

**Affiliations:** ^1^ Department of Radiology and Gruss Magnetic Resonance Research Center Albert Einstein College of Medicine, Montefiore Medical Center Bronx New York USA; ^2^ Laureate Institute for Brain Research Tulsa Oklahoma USA; ^3^ Computer Science Department of Georgia State University Atlanta Georgia USA; ^4^ Tri‐Institutional Center for Translational Research in Neuroimaging and Data Science (TReNDS) Atlanta Georgia USA; ^5^ Maryland Psychiatric Research Center, Department of Psychiatry University of Maryland School of Medicine Baltimore Maryland USA

**Keywords:** brain dynamics, computer vision, dynamic brain map, fMRI, spatiotemporal dense prediction, vision transformer, weakly supervised learning

## Abstract

Modeling dynamic neuronal activity within brain networks enables the precise tracking of rapid temporal fluctuations across different brain regions. However, current approaches in computational neuroscience fall short of capturing and representing the spatiotemporal dynamics within each brain network. We developed a novel weakly supervised spatiotemporal dense prediction model capable of generating personalized 4D dynamic brain networks from fMRI data, providing a more granular representation of brain activity over time. We developed a model that leverages the vision transformer (ViT) as its backbone, jointly encoding spatial and temporal information from fMRI inputs using two different configurations: space–time and sequential encoders. The model generates 4D brain network maps that evolve over time, capturing dynamic changes in both spatial and temporal dimensions. In the absence of ground‐truth data, we used spatially constrained windowed independent component analysis (ICA) components derived from fMRI data as weak supervision to guide the training process. The model was evaluated using large‐scale resting‐state fMRI datasets, and statistical analyses were conducted to assess the effectiveness of the generated dynamic maps using various metrics. Our model effectively produced 4D brain maps that captured both inter‐subject and temporal variations, offering a dynamic representation of evolving brain networks. Notably, the model demonstrated the ability to produce smooth maps from noisy priors, effectively denoising the resulting brain dynamics. Additionally, statistically significant differences were observed in the temporally averaged brain maps, as well as in the summation of absolute temporal gradient maps, between patients with schizophrenia and healthy controls. For example, within the Default Mode Network (DMN), significant differences emerged in the temporally averaged space–time configurations, particularly in the thalamus, where healthy controls exhibited higher activity levels compared to subjects with schizophrenia. These findings highlight the model's potential for differentiating between clinical populations. The proposed spatiotemporal dense prediction model offers an effective approach for generating dynamic brain maps by capturing significant spatiotemporal variations in brain activity. Leveraging weak supervision through ICA components enables the model to learn dynamic patterns without direct ground‐truth data, making it a robust and efficient tool for brain mapping. Significance: This work presents an important new approach for dynamic brain mapping, potentially opening up new opportunities for studying brain dynamics within specific networks. By framing the problem as a spatiotemporal dense prediction task in computer vision, we leverage the spatiotemporal ViT architecture combined with weakly supervised learning techniques to efficiently and effectively estimate these maps.

## Graphical abstract

Overview of the proposed spatiotemporal dense prediction framework. The model takes 4D fMRI data as input and generates dynamic brain maps that evolve over time. The input is first patchified into a sequence of spatiotemporal tokens. A Vision Transformer (ViT) encoder is used to model complex spatial and temporal dependencies across these tokens. We implement and compare two encoder variants: (1) a space‐time encoder, which jointly attends to spatial and temporal tokens within a unified self‐attention mechanism, and (2) a sequential encoder, which applies separate attention over spatial and temporal dimensions. The encoder output is passed to a lightweight CNN‐based decoder head that reconstructs dense spatiotemporal predictions (4D dynamic maps). Weak supervision is incorporated during training via ICA‐derived components, which guide learning through a soft loss function. This architecture enables learning of subject‐specific, temporally dynamic brain activity patterns from weak supervision.

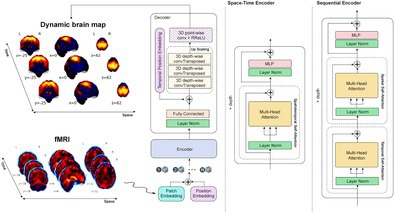

## 1 Introduction

The human brain, an incredibly complex system, consists of nearly one hundred billion neurons, each forming tens of thousands of unique connections with other neurons (Bear et al. 2020; Hawrylycz et al. 2012; Sporns 2022). This forms complex computational and memory networks that exhibit non‐linear dynamics underpinning essential functions such as cognition, memory, emotion, and perception. This dynamic behavior is key to shaping conscious experience and understanding human cognition and consciousness (Liégeois et al. 2019; Raut et al. 2020; Bruining et al. 2020).

Advances in neuroimaging, particularly functional magnetic resonance imaging (fMRI), have significantly transformed computational neuroscience by enabling the simultaneous recording of blood oxygenation level dependence (BOLD) signal activity across the entire brain (Zhang, Wang, and Liu 2024; Marino and Mantini 2024). Many analytical and modeling techniques have been developed to investigate brain dynamics using sequential neural data. One widely used method is based on co‐activation patterns (CAPs), which identify transient brain patterns that contribute to the formation of resting‐state networks (Liu et al. 2018; Zhang, Lin, et al. 2024). CAPs provide an effective method for identifying recurring neural configurations but struggle to resolve meaningful patterns when functionally distinct brain states exhibit significant temporal overlap, potentially limiting their sensitivity in capturing complex or concurrent neural processes (Li et al. 2021; Matsui et al. 2022; Iraji et al. 2022).

Another prominent approach is sliding window correlation (SWC), commonly used to investigate dynamic changes in regional connectivity. This method involves calculating pairwise correlation or covariance matrices within a specified time window across successive time points (Vergara et al. 2019; Shakil et al. 2016). While it is effective for tracking the temporal evolution of functional connectivity, the choice of window length can significantly influence the results. Newer models attempt to relax this constraint (Faghiri et al. 2021). However, overlapping windows may introduce correlated noise and redundancy, complicating the distinction between true dynamic changes and artifacts in the data (Hindriks et al. 2016; Savva et al. 2020, 2019).

Similarly, phase synchrony (PS) has been used to measure coherence and phase coupling between neural components, providing insights into the synchrony between brain regions (Honari et al. 2021; Omidvarnia et al. 2016). This method is well‐suited for capturing the temporal coordination of neural activity; however, it is primarily focused on phase relationships and may not fully capture the amplitude variations (Sadaghiani et al. 2012; Zarghami et al. 2020; Honari and Lindquist 2022). Switching linear dynamical systems (SLDS) are another modeling approach used to represent nonlinear dynamics by approximating complex systems through transitions between multiple linear regimes (Li, Li, et al. 2024). These models are flexible and can represent nonlinear behaviors in brain activity, but the need for multiple discrete states to approximate a single nonlinear vector field can make the models difficult to interpret. The number of latent dimensions required for each regime further complicates the visualization and interpretation of results (Karniol‐Tambour et al. 2024).

Although many methods focus on temporal variations across fixed spatial nodes, recent work emphasizes the importance of capturing spatial variability within functional domains for a more comprehensive understanding of brain activity (Iraji et al. 2020). Quasi‐periodic patterns (QPPs) capture reliable spatiotemporal patterns in low‐frequency neural activity (Fransson and Strindberg 2023; Thompson et al. 2014). This approach is valuable for uncovering recurring dynamics over time, offering insights into both temporal and spatial interactions in brain function. However, QPPs are primarily focused on low‐frequency activity, potentially limiting their ability to detect higher‐frequency events critical for understanding brain dynamics (Abbas 2019; Belloy et al. 2018; Seeburger et al. 2024). Hierarchical models that integrate high‐order spatial independent component analysis (ICA) reveal the fluidity of spatial associations, distinguishing functional homogeneity and stability at lower levels from greater spatial variability at higher levels (Iraji, Deramus, et al. 2019; Iraji, Fu, et al. 2019). Windowed ICA approaches provide even more flexibility, allowing spatially varying maps for each brain network (Iraji, Deramus, et al. 2019).

Although each method offers distinct advantages, a comprehensive understanding of brain dynamics requires analyzing both the temporal evolution of individual networks and the interactions between different brain regions over short timescales. The generation of dynamic brain networks can be reframed as a computer vision problem, using UNet‐style models to capture spatially evolving patterns in brain data (Kazemivash and Calhoun 2020, 2022a). These generated maps have been effective in distinguishing between schizophrenia and control groups, demonstrating their potential for identifying subtle alterations in brain activity (Kazemivash and Calhoun 2022b; Kazemivash et al. 2023). While these innovative methods advance the creation of dynamic brain maps, the development of robust parcellation techniques that yield detailed, temporally resolved maps remains a critical challenge in the field.

In this study, we present a novel model that addresses three challenges in the analysis of dynamic brain networks. First, the model introduces a soft brain parcellation technique that produces high‐resolution 4D brain maps. Second, it functions as a dynamic estimation tool, capturing and representing distinct spatiotemporal activity patterns across multiple brain networks over time. Third, it overcomes the absence of ground‐truth data by incorporating spatially constrained windowed ICA components (Iraji et al. 2024) as weak supervision to initialize the training. Although we use linear ICA‐derived components as priors, our model is designed to also capture nonlinear spatial dependencies and temporal dynamics in brain activity patterns, enabling a more expressive, flexible, and data‐driven representation of functional networks. Finally, we assess the medical relevance and performance of the generated 4D maps to highlight the practical impact of our approach.

## 2 Related Concepts

In this section, we explore key concepts central to our approach, including weakly supervised learning, spatiotemporal dense prediction, and brain parcellation. Understanding these foundational ideas is essential for grasping the methods and objectives of our research on dynamic brain mapping.

### 2.1 Weakly Supervised Learning

Weakly supervised learning approaches allow predictive models to be trained using datasets that lack precise, fully labeled examples. Instead of relying on exact labels for every data point, these methods can handle noisy, ambiguous, or incomplete data (Murphy 2022; Ahuja et al. 2022). One common scenario in weakly supervised learning is label uncertainty, where each training instance is associated with a distribution of possible labels rather than a single definitive label. In such cases, training is performed by minimizing the cross‐entropy between the true label distribution and the model's predicted distribution, a technique known as label smoothing. This method can regularize the model by replacing hard labels with softer, probabilistic versions (Müller et al. 2019; Gong et al. 2024; Liu et al. 2022).

Another method in weakly supervised learning is multiple instance learning (MIL). In MIL, training data is grouped into sets or “bags” where only a label for the entire bag is available, but not for individual instances within the bag (Lv et al. 2023; Yuan et al. 2021; Cui et al. 2024). Distant supervision is yet another strategy in this framework, where labels are inferred from external knowledge sources, such as databases (Qi et al. 2024; Yao et al. 2021; Lin et al. 2022). Label‐noise learning (LNL), which addresses issues caused by incorrect or noisy labels from sources like human error, data uncertainty, or subjective labeling criteria, along with other methods, enables machine learning models to be trained effectively even when ground‐truth data is sparse, incomplete, or noisy (Lu et al. 2023; Han et al. 2020; Rokham et al. 2023, 2020). In the era of big data, where obtaining fully labeled datasets is often impractical, weakly supervised learning has become an essential approach.

### 2.2 Spatiotemporal Dense Prediction

In computer vision, dense prediction is a task of pixel‐wise or voxel‐wise predictions, where the model generates outputs at the level of individual picture or volume elements, rather than at higher levels of abstraction (Yang, Jiang, et al. 2024; Yang, Yuan, et al. 2024; Xia et al. 2024). Examples of dense prediction tasks include semantic (Alexandropoulos et al. 2024; Sung et al. 2024), panoptic (de Geus and Dubbelman 2024; Sun et al. 2024), and instance segmentation (Kalluri et al. 2024; Wei et al. 2024), optical flow estimation (Luo, Li, et al. 2024; Luo, Luo, et al. 2024), depth estimation (Li, Wang, et al. 2024; Ke et al. 2024), and even image reconstruction (Szymanowicz et al. 2024; Charatan et al. 2024).

The dense prediction models need to accurately capture both spatial and temporal information to produce coherent spatiotemporal representations (Weng et al. 2024; Nguyen et al. 2024). To address these challenges, recent techniques have been developed that aggregate flow‐guided features, leverage sequence modeling, and apply heuristic approaches to model temporal variations. These methods aim to exploit redundancies and correlations across different time points, improving both predictive accuracy and efficiency (Arnab et al. 2021).

### 2.3 Brain Parcellation

Brain parcellation refers to the segmentation of the brain into distinct regions that are assumed to be functionally or structurally distinct (Moghimi et al. 2022). The simplest approaches are atlas‐based methods that rely on predefined anatomical templates with rigid boundaries (i.e., hard parcellation) and therefore face limitations due to individual variability in brain size, shape, and folding, as well as the computational demands of spatial registration (Fan et al. 2016; Auzias et al. 2016).

Alternatively, segmentation approaches use functional connectivity (Lawrence et al. 2021) parcellation that clusters voxels based on their connectivity profiles, providing an individual‐level data‐driven representation of brain organization (Eickhoff et al. 2015). Among connectivity‐based approaches, independent component analysis (ICA), a soft parcellation technique (i.e., allowing voxels to contribute to multiple networks with varying weights), is widely employed (Calhoun and Adali 2012). ICA extracts independent spatial maps and captures the temporal contribution of these maps through corresponding time courses. However, reducing these temporal contributions to scalar values per volume may oversimplify the underlying complexity of brain activity (Iraji et al. 2020; Calhoun et al. 2009, 2015). Consequently, there remains a critical gap in the development of advanced dynamic brain parcellation methods capable of fully capturing the intricate temporal dynamics present in neural data and generating spatiotemporally evolving brain activity patterns that accurately reflect the continuous nature of functional brain processes.

## 3 Methods

**FIGURE 1:**
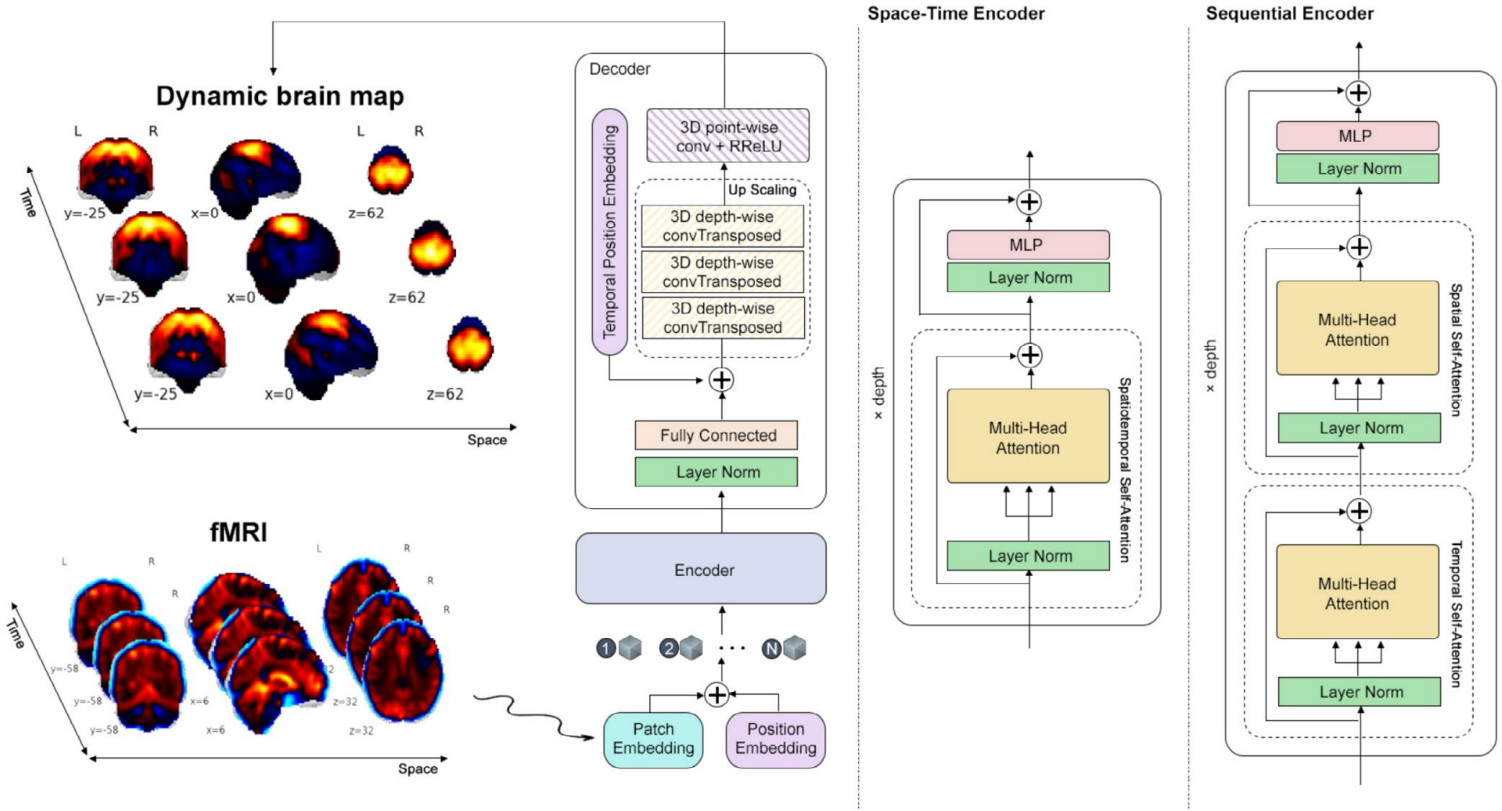
Overview of the proposed spatiotemporal dense prediction framework. The model takes 4D fMRI data as input and generates dynamic brain maps that evolve over time. The input is first patchified into a sequence of spatiotemporal tokens. A Vision Transformer (ViT) encoder is used to model complex spatial and temporal dependencies across these tokens. We implement and compare two encoder variants: (1) a space–time encoder, which jointly attends to spatial and temporal tokens within a unified self‐attention mechanism, and (2) a sequential encoder, which applies separate attention over spatial and temporal dimensions. The encoder output is passed to a lightweight CNN‐based decoder head that reconstructs dense spatiotemporal predictions (4D dynamic maps). Weak supervision is incorporated during training via ICA‐derived components, which guide learning through a soft loss function. This architecture enables the learning of subject‐specific, temporally dynamic brain activity patterns from weak supervision.

Our model generates dynamic patterns of brain networks by employing two distinct configurations independently: a space–time encoder and a sequential encoder, both of which utilize the self‐attention mechanism (updating features based on relationships within the input sequence only) to encode spatial and temporal information using different strategies, as shown in Figure 1. Additionally, we propose a method to tackle the challenge of missing ground‐truth data when generating dynamic brain maps.

We utilize a vision transformer as the backbone of our model. First, we patchify the input fMRI data (i.e., Dividing the 3D spatial volume, height by width by depth, into smaller cubic pieces called patches, at each time point), $x\in {\mathbb{R}}^{h\times w\times d\times t}$, into a sequence of tokens $\tilde{z}\in {\mathbb{R}}^{t\times p\times e}$. More precisely, we extract p non‐overlapping 3D patches $s_{i}$ from the spatial dimensions, apply a linear projection π, and then flatten them into 1D feature vectors, or tokens (a numeric representation of each patch information), $z_{i}\in {\mathbb{R}}^{e}$. Additionally, a learnable positional embedding, $\mathrm{pe}\in {\mathbb{R}}^{t\times p\times e}$, is added to the tokens to retain positional information, addressing the permutation invariance of self‐attention module within the transformer. So we have ${\tilde{z}}_{t}$ representing a token for a timepoint t as follows:

(1) $${\tilde{z}}_{t}=\left[\pi \left(s_{1}^{t}\right),\cdots ,\pi \left(s_{p}^{t}\right)\right]+{\mathrm{pe}}_{t}$$

### 3.1 Space–Time Configuration

In our first scenario, the encoder architecture is designed to process all extracted patches from all spatial locations across all time points simultaneously. By feeding the entire sequence of spatiotemporal tokens into the encoder, the model can effectively learn complex spatial structures while capturing temporal dependencies. To achieve this, we employ an encoder equipped with a spatiotemporal self‐attention module, which enables the model to weigh the importance of different tokens dynamically. This mechanism allows the encoder to jointly encode both spatial and temporal information, facilitating the extraction of meaningful dynamic patterns across brain volumes over time.

The encoder consists of a stack of L transformer layers, each incorporating key components such as multi‐head self‐attention (MHSA), layer normalization (LN), and a multi‐layer perceptron (MLP) with two fully connected layers. Additionally, the MLP includes dropout for regularization and employs randomized leaky rectified linear unit (RReLU) activation to enhance model expressivity. This structure ensures that the encoder effectively models complex interactions within the data, capturing both local and global spatiotemporal dependencies.

(2) $${\tilde{z}}^{\ell +1}=\text{MHSA}\left(\mathrm{LN}\left({\tilde{z}}^{\ell }\right)\right)+{\tilde{z}}^{\ell }$$

(3) $${\tilde{z}}^{\ell +1}=\mathrm{MLP}\left(\mathrm{LN}\left({\tilde{z}}^{\ell +1}\right)\right)+{\tilde{z}}^{\ell +1}$$

The attention mechanism enables the model to selectively focus on relevant features within the input data by weighting the importance of different tokens (spatiotemporal features). This capability allows the model to capture dynamic patterns more effectively, which is particularly crucial for modeling complex temporal dependencies. Moreover, the attention mechanism is defined as follows, where the queries Q, keys K, and values V are linear projections of the input, with $Q,K,V\in {\mathbb{R}}^{p\times e}$, and e representing the embedding dimension. All spatiotemporal tokens extracted from input data are then passed through the transformer encoder to capture dynamic patterns:

(4) $$\text{Attention}\left(Q,K,V\right)=\text{Softmax}\left(\frac{Q.K^{T}}{\sqrt{e_{k}}}\right)V$$

### 3.2 Sequential Encoders Configuration

In our second configuration, we design an encoder architecture that separately models spatial and temporal dependencies using two distinct transformer‐based modules: a temporal attention encoder and a spatial attention encoder. Unlike the first configuration, which employs a unified spatiotemporal self‐attention mechanism, this approach explicitly factorizes spatial and temporal feature extraction to enhance structured representation learning.

First, we process the fMRI data by extracting spatial patches at each time point, resulting in a sequence of spatial tokens. The input token tensor, initially shaped as $\tilde{z}\in {\mathbb{R}}^{t\times p\times e}$, is permuted to $\tilde{z}\in {\mathbb{R}}^{p\times t\times e}$ so that temporal relationships can be effectively captured. This reshaped tensor is then fed into the temporal encoder, which applies multi‐headed self‐attention (MHSA) across the time dimension while preserving spatial structure. By doing so, the temporal encoder learns dependencies across different time points, allowing the model to capture dynamic patterns in brain activity.

Next, the output from the temporal encoder is reshaped back to its original form, $\tilde{z}\in {\mathbb{R}}^{t\times p\times e}$, and passed into the spatial encoder. This module applies self‐attention over spatial tokens at each time step, refining spatial feature representations while incorporating the temporal context previously learned. The embedding dimensionality remains consistent between the temporal and spatial encoders to ensure seamless information propagation and avoid representational discrepancies. By leveraging separate attention modules for spatial and temporal encoding, this two‐stage strategy allows the model to effectively capture dynamic spatiotemporal dependencies while maintaining computational efficiency.

### 3.3 Decoder Head

Unlike the encoder, which is built on the ViT architecture, the decoder head is designed with a series of components. It consists of a layer normalization, followed by a fully connected layer, a fixed sine‐cosine positional encoding to represent the time‐point indices, and a sequence of 3D depth‐wise transposed convolution operator (ConvTranspose) layers with various kernel sizes of 7, 5, and 9, respectively. The final ConvTranspose layer has a dilation rate of 2. The decoder also includes a point‐wise Conv3D layer, with RReLU serving as the activation function. The output of the decoder corresponds to the predicted spatiotemporal brain maps.

### 3.4 Customized Loss Function

Selecting an appropriate loss function that simultaneously preserves the global structure of the data while capturing dynamic patterns is a challenging task. To address this, we utilize a combination of photometric and perceptual losses to guide the network's training, as formulated below:

(5) $$ℒ=E_{y,\hat{y}∼U}\;\left[\frac{\log\left(\cosh\left(y-\hat{y}\right)\right)}{\max\;\left(\epsilon ,\text{SSIM}\left(y,\hat{y}\right)\right)}\right]$$

(6) $$\text{SSIM}\left(y,\hat{y}\right)=\frac{\left(2\mu_{y}\mu_{\hat{y}}+C_{1}\right)\left(2\sigma_{y\hat{y}}+C_{2}\right)}{\left(\mu_{y}^{2}+\mu_{\hat{y}}^{2}+C_{1}\right)\left(\sigma_{y}^{2}+\sigma_{\hat{y}}^{2}+C_{2}\right)}$$

Here, y represents the prior (weak supervision), and $\hat{y}$ corresponds to the model's prediction, while SSIM denotes the structural similarity index. $E_{y,\hat{y}∼U}$ represents the expectation over all samples in the trainset (true y and output $\hat{y}$), which, given that they are assumed to be uniformly drawn, is practically computed as an average over a batch in our training phase. The parameter ϵ=0.001 is used to prevent division by zero or negative values in the denominator and $C_{1},C_{2}$ are small constants to stabilize the division. The $\log\left(\cosh\left(.\right)\right)$ function serves as a robust regression loss, similar to mean squared error (MSE), but less prone to being influenced by outliers. Similar to the Huber loss, the $\log\left(\cosh\left(\cdot \right)\right)$ function tries to strike a balance between L1 loss for extreme values and L2 loss for the rest, providing robustness to large residuals. However, unlike the Huber loss—which is only piecewise differentiable and has a non‐smooth point at the transition threshold—$\log\left(\cosh\left(\cdot \right)\right)$ is continuously differentiable everywhere. This smoothness enables more stable and consistent gradient updates, which can improve optimization dynamics and convergence speed and reliability. Additionally, we observe empirically that incorporating SSIM loss creates more opportunities for improving prediction quality.

### 3.5 Weak Supervision

One of the main challenges in our research is the lack of ground‐truth labels, which we addressed by utilizing a windowed version of spatially constrained ICA (Iraji et al. 2024), a powerful semi‐blind source separation technique (Lin et al. 2010). This method enables us to extract independent components (ICs), each representing a distinct brain network, which we used as weak supervision to guide the training of our model. Specifically, we applied spatially constrained ICA in a sliding‐window fashion to extract temporally localized independent components (ICs) that serve as coarse spatiotemporal priors. This provides a robust and computationally tractable initializer, using a linear unmixing approach. We incorporated the ICs as soft prior during training, encouraging the model to align with their general spatial topology without enforcing strict voxel‐level correspondence. Unlike ICA, which is restricted to linear unmixing, our deep learning model learns a nonlinear spatiotemporal mapping capable of capturing complex and temporally coherent brain activity. As a result, the model produces smooth, denoised, and interpretable 4D brain activity maps that generalize beyond the noisy supervision provided by ICA.

Our method leverages constrained ICA by using the NeuroMark‐fMRI‐1.0 template (Du et al. 2020), which consists of reproducible independent components derived from the Genomic Superstructure Project (GSP) and Human Connectome Project (HCP) datasets. These group‐level constrained windowed ICs were computed by spatially aligning the correlated components from more than 800 healthy control fMRI datasets. The NeuroMark template provides a reference for calculating subject‐specific ICs using a multi‐objective optimization strategy as follows:

(7) X=AS

(8) $$\begin{array}{l} \max\;J\left(w_{i}\right)\propto {\left\{E\left[G\left(C_{i}\right)\right]-E\left[G\left(v\right)\right]\right\}}^{2}\\ s.t.\;\varphi \left(w_{i}^{T}x,R_{i}\right)-\epsilon \leq 0,E\left[C_{i}^{2}\right]-1=0 \end{array}$$

In this context, $X\in {\mathbb{R}}^{t\times n}$ denotes the input fMRI data matrix with t time points and voxel size of n, modeled as X=AS, where $A\in {\mathbb{R}}^{t\times m}$ is the mixing matrix containing the temporal profiles of the components as columns, and $S\in {\mathbb{R}}^{m\times n}$ contains the spatially independent sources as rows. To estimate the sources, we compute an unmixing matrix $W\in {\mathbb{R}}^{m\times t}$ such that S=WX. Each spatial component $C_{i}\in {\mathbb{R}}^{1\times n}$ is obtained as $C_{i}=w_{i}^{⊤}X$, where $w_{i}\in {\mathbb{R}}^{t}$ is the *i*‐th row of W, and corresponds to the inverse of the associated time course in A. The term $J\left(w_{i}\right)$ estimates the negentropy of the *i*‐th source $C_{i}$, with $w_{i}$ as its corresponding unmixing vector. $E\left[.\right]$ stands for the expectation, while $G\left(.\right)$ is a non‐quadratic function. The variable v is a Gaussian random variable with a mean of zero and unit variance. The function φ measures the similarity (Pearson correlation) between the estimated source $C_{i}$ and its normalized reference $R_{i}$, with ϵ acting as a threshold to control the separation accuracy between the independent component and the reference signal. We utilize spatially constrained ICA on fMRI data by implementing overlapping windows, with a window size of 30 and a stride of 1, to extract a sequence of independent components. The choice of a 30‐point window reflects a heuristic balance between capturing dynamic temporal changes and maintaining stability in component estimation. This approach offers advantages in extracting brain networks compared to other methods, though it remains a linear technique. Additionally, spatially constrained windowed ICA method is sensitive to noise, and in cases of low signal‐to‐noise ratio, the accuracy of component separation may be affected. In the windowed ICA configuration, it is also assumed that the statistical properties of the sources remain constant, an assumption that may not always hold. Despite these limitations, spatially constrained windowed ICA can serve as a suitable weak prior for a deep learning model, as we propose in this work. Alternative approaches also exhibit various limitations. For example, anatomic atlas‐based methods rely on fixed, predefined templates that overlook individual variability in brain organization. Clustering‐based techniques typically require pre‐specifying the number of clusters, are sensitive to initialization, and may lead to spatially fragmented regions. Graph‐based methods are highly dependent on graph construction strategies and often lack spatial contiguity. While IVA‐based approaches are effective in modeling inter‐subject variability, they are computationally expensive and are typically estimated from group data rather than from a single subject.

## 4 Experiment

We implemented a straightforward experimental setup for our research. Our study utilized a subset of 508 fMRI datasets, which included data from the MPRC (Adhikari et al. 2019), FBIRN (Potkin and Ford 2009), and COBRE (Mayer et al. 2013) datasets. The demographic information for this subset is outlined in Table 1. To reduce computational demands, we uniformly sampled time points from the fMRI data at 10‐timepoint intervals, resulting in a total of 10 time points instead of using the full dataset. We also applied a Gaussian filter for image smoothing, with a standard deviation of σ=6 set for Gaussian kernel, followed by z‐scoring to ensure proper normalization of the data. The model configuration included an embedding dimension of 96, 6 attention heads, a depth of 1, an attention dropout rate of 0.4, and an encoder dropout rate of 0.3, with a patch size of 5. Training was conducted on two A40 GPUs, each equipped with 45 GB of memory, using a batch size of 2. We utilized the Adam optimizer with a learning rate of 0.01 and a weight decay of 0.1, running for a total of 150 epochs and implementing an early stopping strategy to prevent overfitting, with a threshold of $\delta =10^{-5}$.

**TABLE 1:** Demographic information.

Diagnostic	Sex		Age	Race		
	Male	Female	Mean ± SD	American	European	Other
Control	185	130	38.40 ± 12.73	192	65	58
Schizophrenia	154	39	38.61 ± 13.29	112	44	37

### 4.1 Qualitative Metrics

**FIGURE 2:**
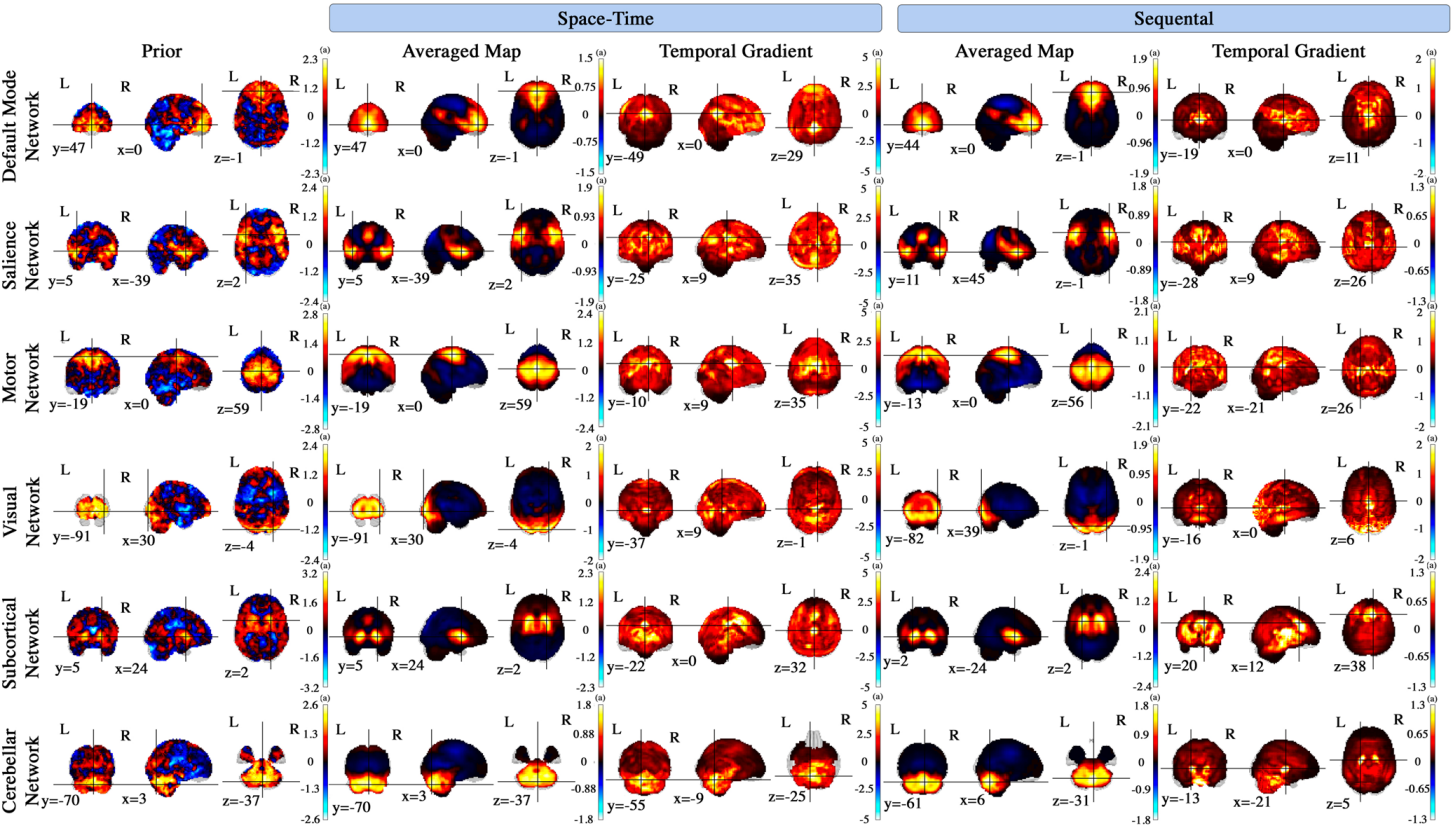
Model‐generated dynamic brain maps within the Default Mode, Salience, Motor, Visual, Subcortical, and Cerebellar networks, averaged over time for a randomly selected test subject. The figure includes the summation of absolute temporal gradient maps and prior maps, comparing outputs from both the Space–Time encoder and Sequential encoder configurations.

We present qualitative comparisons between the dense visual representations learned by our model and the extracted sequence of ICA components (used for weak supervision) across different networks, employing both space–time and sequential encoder configurations, as shown in Figure 2. The dynamic patterns of brain activities observed in the generated maps exhibit smooth transitions in both space and time, with noticeable fluctuations in activation weights (scores) across different time points. These transitions highlight the model's capability to capture temporal continuity, resulting in more plausible representations of brain activity. The generated maps show a strong alignment with known brain atlases, particularly in the spatial localization of functional regions, reinforcing the model's accuracy in learning meaningful spatial features. Moreover, we provide additional evidence of the model's ability to capture dynamic patterns over time by calculating the summation of absolute temporal gradients, approximated using the forward difference approach. This metric quantifies the temporal changes in brain activity, offering a complementary view of how the model captures both gradual and abrupt transitions across time points. The temporal gradients underscore the model's sensitivity to changes in activation dynamics, further validating its capacity to generate temporally consistent and biologically meaningful representations.

**FIGURE 3:**
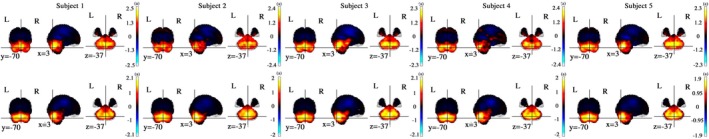
Generated maps for five example subjects at the 5th time point illustrate inter‐subject variations, with some observable differences in morphology, scale, spatial displacement, and activation intensity of active regions. The top row shows the space–time configuration, while the bottom row shows the sequential encoder configuration.

Additionally, the subject‐specific maps in the cerebellar network at the 5th time point, as illustrated in Figure 3, show examples of individual‐level variations captured by the model. These observations indicate that the model can reflect differences across subjects, although they should be interpreted as illustrative rather than conclusive evidence of population‐level heterogeneity. Furthermore, the model's robustness is evidenced by its ability to effectively denoise highly noisy prior (weak supervision), refining them into clearer, more interpretable dynamic brain maps. This denoising capability not only improves the signal‐to‐noise ratio but also enhances the interpretability of the spatiotemporal patterns, facilitating a deeper understanding of the underlying neural processes. Overall, the model translates raw, noisy data into coherent, higher‐resolution representations that capture global patterns of brain activity over time, while also allowing for the depiction of possible individual‐level differences within each brain network.

**FIGURE 4:**
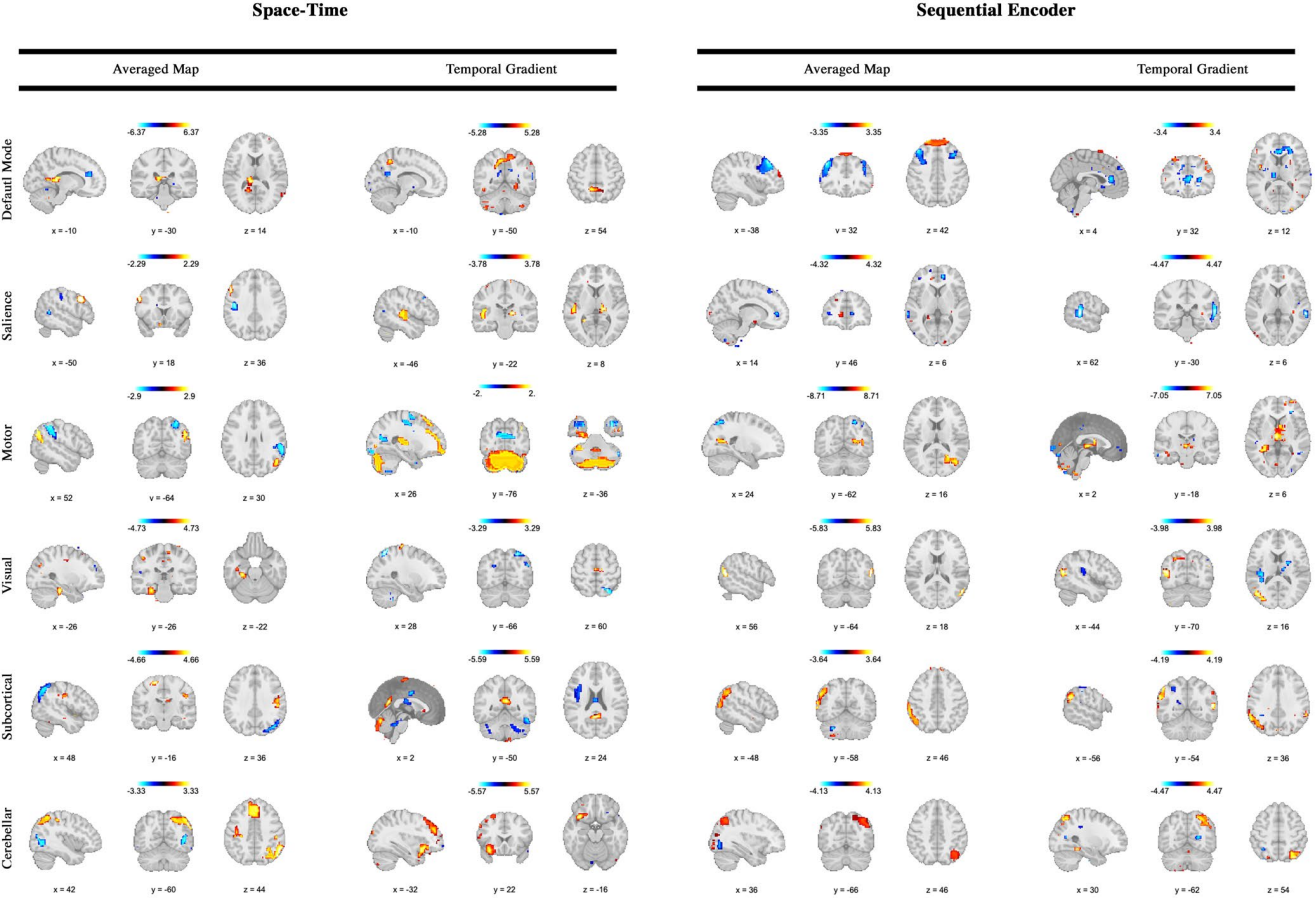
Group‐level differences across multiple regions are highlighted in both the averaged maps and the sum of temporal gradients, assessed using a two‐sample t‐test with FDR correction and masked at p≤0.05 across the default mode, salience, motor, visual, subcortical, and cerebellar networks. The maps display $-\log\left(p\_\text{value}\right)\times \mathrm{sign}\left(t\_\text{value}\right)$, emphasizing the relevance of the generated dynamic maps to our understanding of schizophrenia. Widespread group differences are observed across several brain regions, with schizophrenia associated with both spatial and temporal changes. While healthy controls (HCs) generally exhibit greater hyperactivity and variations, schizophrenia demonstrates increased activity and variation in specific regions. Notably, these changes often exhibit structured patterns that do not strictly align with regions strongly contributing to the networks, suggesting the presence of transient states or functional connectivity across networks. The left two columns illustrate the space–time configuration, whereas the right two columns present the sequential encoder configuration, highlighting differences between the two scenarios.

To assess the clinical relevance of the generated dynamic brain maps, we conducted a series of experiments to explore their potential for differentiating between healthy controls (HC) and individuals with schizophrenia (SCZ). The dynamic brain maps, generated by our model for all subjects, included both HC and SCZ groups. In the first experiment, we averaged the dynamic maps across time and applied a mask to extract brain voxels, $\mathrm{HC}\in {\mathbb{R}}^{315\times 68,235}$ and $\mathrm{SCZ}\in {\mathbb{R}}^{193\times 68,235}$. A voxel‐wise t‐test was then performed to identify regions with significant differences between the two groups, and the resulting *p*‐values were corrected for multiple comparisons using false discovery rate (FDR) correction at a significance level of p≤0.05. This analysis enabled the detection of specific brain regions that exhibit altered dynamics in SCZ, potentially revealing biomarkers for the disorder. In the second experiment, we applied the same t‐test to the TG maps, which capture voxel‐wise differences between consecutive time‐points, to assess group differences in dynamic brain activity. This approach allows us to study variations in dynamic brain patterns, as shown in Figure 4, and their potential association with the pathophysiology of schizophrenia. These findings lay a foundation for further exploration of their relevance in clinical observations and future research on diagnostic and therapeutic strategies.

### 4.2 Quantitative Metrics

We quantitatively assess the model's capacity to generate plausible dense representations using an indirect standard approach. This evaluation focuses on several key metrics, including active region localization accuracy, visual fidelity, consistency with expected patterns, and regional homogeneity, which is quantified by the correlation of voxel time‐series within each region of interest (ROI). We perform these assessments on both the generated maps and the priors, averaging results over time, while applying a threshold of ≥80% specifically to the mean intersection over union (mIOU) and homogeneity (Hgt) metrics, as detailed in Table 2. Here, mIOU refers to the mean intersection over union, which quantifies spatial overlap between the predicted and reference activation regions; Hgt denotes the homogeneity of within‐region temporal variations, capturing region‐wise consistency in dynamic signal fluctuations; and mARE represents the mean absolute relative error, which measures the relative voxel‐wise deviation between predicted and reference maps. SSIM signifies the structural similarity index, reflecting perceptual and structural alignment between spatial patterns.

**TABLE 2:** Quantitative metrics on quality of generated dynamic maps.

	Networks	mARE ↓	mIOU ↑	SSIM ↑	Hgt ↑
Space–Time	Default Mode	0.12	0.77	**0.87**	0.77
	Salience	0.17	0.71	0.79	0.79
	Motor	0.14	0.73	0.86	0.77
	Visual	0.15	0.77	0.84	0.81
	Subcortical	0.15	0.77	0.81	0.72
	Cerebellar	**0.1**	**0.81**	0.86	**0.84**
Sequential Enc	Default Mode	0.15	0.73	**0.85**	0.7
	Salience	0.21	0.69	0.73	0.63
	Motor	0.17	0.72	0.8	0.74
	Visual	0.16	0.77	0.8	0.72
	Subcortical	0.17	0.74	0.75	0.66
	Cerebellar	**0.13**	**0.79**	0.82	**0.79**

*Note:* Bold values indicate the best result for each metric.

The quantitative results reveal several interesting patterns across networks and encoding schemes. The Cerebellar network stands out with the best overall performance, achieving the lowest mARE and highest Hgt in both Space–Time (mARE: 0.10, Hgt: 0.84) and Sequential Enc (mARE: 0.13, Hgt: 0.79), along with competitive mIOU and SSIM scores. In contrast, the Salience network consistently underperforms, with the highest mARE and lowest mIOU, SSIM, and Hgt in both encoding schemes, suggesting challenges in capturing its diffuse and variable activity patterns. The Default Mode network excels in structural similarity, achieving the highest SSIM (0.87 in Space–Time and 0.85 in Sequential Enc), indicating robust reconstruction of its coherent activity patterns. The Visual network demonstrates stable performance, with relatively high mIOU and Hgt scores across both schemes, reflecting the predictable nature of visual dynamics. Notably, Space–Time encoding generally results in lower mARE values, highlighting better reconstruction accuracy, while Sequential Enc slightly outperforms in mIOU for the Cerebellar and Subcortical networks, suggesting improved spatial overlap capture in specific cases. These findings are consistent with the expected dynamic characteristics of these brain networks.

**FIGURE 5:**
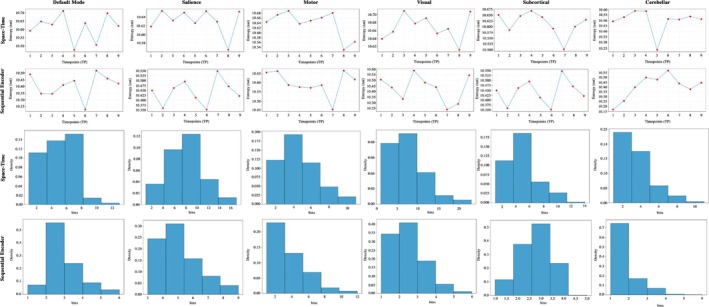
Shannon entropy trends (computed for a single random test subject) and blob density histograms (calculated across the entire test set) for six brain networks: Default mode, salience, motor, visual, subcortical, and cerebellar. These are presented for two encoding schemes: A space–time configuration (rows 1 and 3) and a sequential encoder configuration (rows 2 and 4). The space–time configuration shows a somewhat wider range of entropy fluctuations across networks, indicating slightly more sensitivity to temporal changes, while the sequential encoder configuration exhibits more stable temporal gradients in Shannon entropy, reflecting consistent representations of spatiotemporal dynamics (e.g., DMN entropy ranges from [10.5, 10.7] for space–time and [10.25, 10.50] for sequential encoder). The blob density histograms reveal distinct behaviors across networks in both configurations. For instance, in the sequential encoder scenario, the cerebellar network shows a tighter and more compact distribution, which is also shifted to lower values compared to other networks, suggesting higher spatial consistency across the test set—most subjects exhibit a similar, smaller number of blobs throughout the entire time interval. In contrast, the space–time configuration displays a wider distribution for the cerebellar network, indicating higher variability while also being shifted toward lower values. Additionally, the motor network in the sequential encoder scenario demonstrates a noticeable shift toward lower values, highlighting reduced activity or variation in blob density for this configuration.

Furthermore, we evaluate the model's capacity to represent temporal changes by calculating the Shannon entropy of the temporal gradient maps, capturing changes in information content as an additional indication of the model's dynamic behavior. This metric provides a way to quantify evolving patterns across time and serves as an indicator of the variability captured in the model outputs, as illustrated in the upper row of Figure 5.

Additionally, we utilize the connected components algorithm as an effective method to measure the dynamicity of the generated 4D maps. By applying this algorithm to the thresholded maps at each time point with δ=0.65, the algorithm identifies spatially contiguous clusters of active voxels—referred to as “blobs”—which represent regions undergoing dynamic transitions. By counting the number of connected components (blobs) across time and across different networks for the entire test set, we assess the spatial dispersion and temporal evolution of dynamic activity. This approach is illustrated in lower part of Figure 5. The number of blobs changes over time, reflecting occasional merges and shrinkage of active regions, and serves as a descriptive characterization of the dynamic nature of the model outputs.

### 4.3 Ablation Study

To evaluate the influence of key design decisions in our model in both space–time and sequential encoder configurations, we performed comprehensive ablation studies focused on two critical aspects: the number of tokens and the temporal resolution. These were systematically varied by modifying patch sizes and the number of time points included in the input data. By experimenting with different patch configurations and temporal granularity, we sought to understand how these factors impact the quality of the results. Specifically, we trained separate model instances for each configuration and assessed their performance on the test set using structural similarity index (SSIM) and mean relative absolute error (mRAE) as evaluation metrics. The findings from these experiments, detailed in Tables 3 and 4, provide valuable insights into the trade‐offs associated with tokenization strategies and temporal encoding choices, guiding the optimization of our model's architecture for enhanced accuracy and robustness.

**TABLE 3:** Ablation study on visual quality of generated maps using SSIM.

	Networks	PS = 9	PS = 7	PS = 5
Space–Time	Default Mode	0.37	0.51	0.87
	Salience	0.28	0.49	0.79
	Motor	0.32	0.62	0.86
	Visual	0.27	0.59	0.84
	Subcortical	0.23	0.63	0.81
	Cerebellar	0.3	0.66	0.86
Sequential Enc	Default Mode	0.32	0.44	0.85
	Salience	0.3	0.45	0.73
	Motor	0.29	0.6	0.8
	Visual	0.22	0.57	0.8
	Subcortical	0.17	0.6	0.75
	Cerebellar	0.13	0.64	0.82

**TABLE 4:** Ablation study on dynamicity of generated maps using mRAE for different number of time points (TP).

	Networks	TP = 10	TP = 7	TP = 3
Space–Time	Default Mode	0.12	0.1	0.08
	Salience	0.17	0.14	0.1
	Motor	0.14	0.11	0.09
	Visual	0.15	0.13	0.1
	Subcortical	0.15	0.12	0.1
	Cerebellar	0.1	0.08	0.05
Sequential Enc	Default Mode	0.15	0.12	0.1
	Salience	0.21	0.17	0.14
	Motor	0.17	0.16	0.11
	Visual	0.16	0.13	0.11
	Subcortical	0.17	0.15	0.13
	Cerebellar	0.13	0.1	0.07

Our study reveals several important insights regarding the impact of architectural choices on model performance. Specifically, we observe that increasing the patch sizes within the ViT backbone leads to a modest degradation in the visual fidelity of the generated dense representations. Larger patch sizes reduce the spatial resolution of the input data, thereby limiting the model's ability to capture fine‐grained spatial details. This trade‐off results in a less accurate representation of subtle, local features, which are crucial for high‐quality dynamic brain mapping.

Additionally, we found that using fewer time‐points in the temporal encoding significantly affects the spatiotemporal dynamics of the generated maps. When fewer time‐points are included, the resulting maps exhibit more stationary behavior over time. This is because the reduced temporal granularity leads to less variation between consecutive time‐points, as evidenced by a minimal mRAE between consecutive time‐points, averaged across subjects. Consequently, this lack of variation results in a loss of dynamic information, diminishing the model's ability to capture the intricate temporal fluctuations that are essential for modeling brain activity patterns over time. These findings underscore the importance of both spatial and temporal resolution in accurately capturing the dynamic behavior of brain networks and highlight the need for careful selection of patch sizes and time‐points in spatiotemporal modeling tasks.

## 5 Discussion

Our study presents a new approach to capturing brain dynamics that reveal spatiotemporal variations in shape, size, and regional location of active regions. The averaged maps align with the results from established ICA‐based models and existing brain atlases (Calhoun and Adali 2012; Ding et al. 2016). Furthermore, our model helps to interpret the outcomes by generating denoised maps. Our quantitative metrics, including a low mean mARE and high values for mIOU, SSIM, and homogeneity (shown in Table 2), provide strong evidence of the model's ability to produce plausible maps that preserve essential spatial patterns. In the absence of ground‐truth data, we evaluated the method from multiple perspectives, recognizing that different configurations, such as the space–time and sequential encoders, may capture distinct aspects of the underlying dynamics in the fMRI data. The sequential encoder emphasizes temporal patterns, revealing intriguing new properties of the temporal dynamics, as explored in the clinical application section. On the other hand, the space–time encoder demonstrates stronger quantitative performance, effectively capturing both spatial and temporal dynamics. Together, these configurations provide complementary insights, highlighting the multifaceted nature of brain activity and the value of leveraging multiple perspectives to study its complexity.

Furthermore, the summation of absolute temporal gradient maps (TGs) showcases the model's ability to capture dynamic brain activity, providing insights that extend beyond those available from averaged maps and aligning well with recent findings in computational neuroscience. For instance, elevated variation is detected in the posterior cingulate cortex (PCC) of the default mode network (DMN), a region invisible in averaged maps but known for its role in default mode processing (Fransson and Marrelec 2008). Similarly, the temporal gradient map for the sequential configuration highlights prominent activity in the thalamus—a region intricately connected with the medial prefrontal cortex (mPFC) and functionally conserved across mammalian species, including humans (Zhao et al. 2024; Klein et al. 2010).

Within the salience network, the temporal gradient map for the space–time configuration highlights significant activity in the PCC, a region implicated in the processing of salient events and facial recognition (Leech and Sharp 2014). In the motor network, the model captures the expected elevated activity in the midbrain (Ruchalski and Hathout 2012). Additionally, the sequential encoder configuration reveals prominent activity in the prefrontal cortex, a central area for motor planning, decision‐making, and cognitive control (Grafton and Volz 2019). These observations reflect the interconnected nature of these networks as documented in existing research.

In examining the visual network, the model detects activity in the hippocampus, which aligns with literature suggesting the hippocampus's role in spatial processing and its functional coupling with visual cortical neurons during natural behavior (Turk‐Browne 2019; Haggerty and Ji 2015). In contrast, the sequential encoder configuration highlights dynamic patterns in the thalamus, particularly within the lateral geniculate nucleus (LGN)—a crucial relay center for transmitting visual information from the retina to the primary visual cortex (Saalmann and Kastner 2011; Usrey and Alitto 2015).

In the subcortical network, the sequential encoder configuration's temporal gradient reveals notable variability in the anterior cingulate cortex, a region that serves as the origin of the anterior cingulate‐subcortical circuit, providing input to subcortical structures such as the ventral striatum and other related areas (Bonelli and Cummings 2007; Stevens et al. 2011). Additionally, we observe pronounced variability in the activity scores of the cerebellum in the cerebellar network under the space–time configuration, whereas the sequential encoder configuration shows greater variability in the basal ganglia. The interconnection between these regions is well documented, linking the motor and non‐motor domains of one subcortical system to the corresponding domain in the other. This anatomical connection supports the idea that cerebellar output can influence the input stage of the basal ganglia and vice versa (Bostan et al. 2010; Yoshida et al. 2022).

Figure 5 provides further quantitative evidence of the model's ability to capture dynamic brain patterns. In Figure 5, Shannon entropy values are computed over temporal gradient maps for a random test subject across all networks, including the default mode, salience, motor, visual, subcortical, and cerebellar networks. This entropy analysis highlights the model's sensitivity to variations in temporal dynamics, with the top row representing the space–time configuration and the bottom row the sequential encoder configuration. Higher entropy values across networks indicate the model's capacity to capture complex, fluctuating activity patterns across these functional regions. Figure 5 further explores dynamic brain patterns through changes in blob count in the time interval. These metrics emphasize fluctuations in activity within the 4D maps, providing insights into the spatiotemporal variation captured by each configuration. Notably, the space–time configuration exhibits more pronounced changes in blob characteristics than the sequential encoder configuration, underscoring its enhanced ability to capture and represent dynamic spatial features in brain activity.

Overall, these findings demonstrate the model's robustness in capturing complex, dynamic activity patterns across multiple brain networks, highlighting its potential as a tool for examining the temporal and spatial organization of brain function.

### 5.1 Application to Clinical Dataset

In the DMN, there are indications of potential differences in the temporally averaged space–time configurations, particularly within the thalamus, where healthy controls may exhibit higher activity compared to individuals with schizophrenia. This observation aligns, in part, with studies suggesting that disruptions in information flow to and from the thalamus could contribute to symptoms of schizophrenia (Iraji, Deramus, et al. 2019; Pergola et al. 2015; Anticevic and Halassa 2023). Similarly, the TG map suggests possible differences in the parietal lobules, which could indicate dysconnectivity between the parietal lobe and other brain regions in the disorganization symptoms of schizophrenia (Das et al. 2020). Increased activity in the middle frontal gyrus is also tentatively observed in schizophrenia subjects, potentially supporting hypotheses regarding alterations in this region in the condition (Woodward et al. 2011; Pomarol‐Clotet et al. 2010). Finally, variability in the anterior cingulate cortex (ACC) appears elevated in schizophrenia, resonating with findings that report both hypoactivation and hyperactivation of the ACC. This variability might reflect a hyperactive ACC at rest, which could struggle to further activate under increased task demands, resulting in relative hypoactivity (Adams and David 2007).

In the salience network, temporally averaged space–time maps show a tendency for higher prefrontal cortex activation in healthy controls compared to schizophrenia subjects. This observation is partially consistent with research suggesting that schizophrenia patients often face difficulties with tasks dependent on prefrontal cortical function, such as Continuous Performance (attention), Wisconsin Card Sort (cognitive flexibility), Delayed Response (working memory), and N‐Back (working memory) tasks (Selemon and Zecevic 2015). Additionally, there are hints of greater variability in the left primary auditory cortex (Heschl's gyrus) in healthy controls, which could align with findings linking reduced volume and thickness in this region to auditory hallucinations in schizophrenia (Soler‐Vidal et al. 2022; Doucet et al. 2019). Schizophrenia subjects also seem to show localized activation in a small region of the anterior cingulate and increased variability in the right superior temporal sulcus (STS), a region previously associated with hyperactivation in schizophrenia (Wible et al. 2009).

In the motor network, temporally averaged maps suggest hyperactivation in the right lateral parietal region of the DMN in healthy controls, while schizophrenia subjects show hyperactivation in the right inferior parietal cortex. These observations may correspond with existing evidence of altered connectivity patterns in schizophrenia, where the lateral DMN exhibits decreased connectivity with sensorimotor regions but increased connectivity with heteromodal association areas (Wang et al. 2015). Furthermore, higher variability in cerebellar interactions with motor networks is observed in healthy controls, which could align with studies suggesting disrupted functional associations between cerebellar lobules and motor regions in schizophrenia (Kim et al. 2020). Schizophrenia also appears to be associated with hypoactivation in the precuneus (Forlim et al. 2020) and reduced variability in the thalamus compared to healthy controls.

In the visual network, temporally averaged maps suggest hyperactivation in the posterior hippocampus in healthy controls, aligning with previous findings (Ragland et al. 2017). However, schizophrenia subjects show increased variability in the superior parietal cortex, a region involved in spatial perception, attention, and self‐awareness (Yildiz et al. 2011). Similar to the motor network, hypoactivation of the precuneus is observed in schizophrenia. Additionally, reduced variability is seen in the extrastriate cortex for schizophrenia subjects, a region associated with visual processing abnormalities in the disorder (van der Stelt et al. 2006).

In the subcortical network, individuals with schizophrenia show hypoactivation in the primary motor cortex, which may be linked to motor symptoms arising from disrupted neural circuitry and dysregulated dopamine signaling (Uddin et al. 2010). However, they also exhibit hyperactivation in a region of the parietal lobe. Furthermore, increased variability is seen in the default mode network in healthy controls, while these controls show hyperactivation in the left parietal lobe and greater variability in the angular gyrus under sequential encoder configurations.

Finally, the temporally averaged maps of space–time configuration suggest hyperactivation in the parietal lobes and medial frontal gyrus, regions that are known to be functionally connected (O'reilly et al. 2010; Jobson et al. 2021). Healthy controls also show greater variability in the orbitofrontal cortex (Walton et al. 2017). Sequential encoder configurations indicate hyperactivation in the parietal cortex and heightened variability in the intraparietal cortex in healthy controls compared to schizophrenia subjects. While these trends are intriguing, further investigation is required to better understand the complexities of these network alterations in schizophrenia.

## 6 Conclusion

Our study established a new experimental paradigm for the study of brain dynamics by proposing a novel method to model both spatial and temporal dimensions from input fMRI data and generate dynamic brain maps. This approach enables us to study brain dynamics within different brain networks with a high degree of granularity. By leveraging the spatiotemporal ViT architecture and weakly supervised learning techniques, our model effectively captures the spatiotemporal variations in brain activity, even in the absence of direct ground‐truth data. The model's ability to produce smooth, temporally evolving brain maps from noisy priors offers a promising new tool for neuroimaging research, particularly in understanding complex neurological conditions such as schizophrenia. The significant differences observed between patient and control groups underscore the model's potential for clinical applications, including differential diagnosis and personalized treatment strategies. Ultimately, this work lays the foundation for future advancements in dynamic brain mapping, providing a powerful framework for exploring brain activity in health and disease.

## Data Availability

The data that support the findings of this study are available from the corresponding author upon reasonable request.

## References

[hbm70364-bib-0001] Abbas, A. 2019. Understanding Brain Activity Dynamics Through the Investigation of Quasi‐Periodic Patterns. Emory University.

[hbm70364-bib-0002] Adams, R. , and A. S. David . 2007. “Patterns of Anterior Cingulate Activation in Schizophrenia: A Selective Review.” Neuropsychiatric Disease and Treatment 3, no. 1: 87–101. 19300540 10.2147/nedt.2007.3.1.87 PMC2654525

[hbm70364-bib-0003] Adhikari, B. M. , L. E. Hong , H. Sampath , et al. 2019. “Functional Network Connectivity Impairments and Core Cognitive Deficits in Schizophrenia.” Human Brain Mapping 40, no. 16: 4593–4605. 31313441 10.1002/hbm.24723 PMC6865503

[hbm70364-bib-0004] Ahuja, K. , J. S. Hartford , and Y. Bengio . 2022. “Weakly Supervised Representation Learning With Sparse Perturbations.” Advances in Neural Information Processing Systems 35: 15516–15528.

[hbm70364-bib-0005] Alexandropoulos, S. , C. Sakaridis , and P. Maragos . 2024. “Ovenet: Offset Vector Network for Semantic Segmentation.” In Proceedings of the IEEE/CVF Winter Conference on Applications of Computer Vision, 7407–7418. IEEE.

[hbm70364-bib-0006] Anticevic, A. , and M. M. Halassa . 2023. “The Thalamus in Psychosis Spectrum Disorder.” Frontiers in Neuroscience 17: 1163600. 37123374 10.3389/fnins.2023.1163600 PMC10133512

[hbm70364-bib-0007] Arnab, A. , M. Dehghani , G. Heigold , C. Sun , M. Lučić , and C. Schmid . 2021. “Vivit: A Video Vision Transformer.” In Proceedings of the IEEE/CVF International Conference on Computer Vision, 6836–6846. IEEE.

[hbm70364-bib-0008] Auzias, G. , O. Coulon , and A. Brovelli . 2016. “Marsatlas: A Cortical Parcellation Atlas for Functional Mapping.” Human Brain Mapping 37, no. 4: 1573–1592. 26813563 10.1002/hbm.23121 PMC6867384

[hbm70364-bib-0009] Bear, M. , B. Connors , and M. A. Paradiso . 2020. Neuroscience: Exploring the Brain, Enhanced Edition: Exploring the Brain. Jones & Bartlett Learning.

[hbm70364-bib-0010] Belloy, M. E. , M. Naeyaert , A. Abbas , et al. 2018. “Dynamic Resting State fMRI Analysis in Mice Reveals a Set of Quasi‐Periodic Patterns and Illustrates Their Relationship With the Global Signal.” NeuroImage 180: 463–484. 29454935 10.1016/j.neuroimage.2018.01.075 PMC6093802

[hbm70364-bib-0011] Bonelli, R. M. , and J. L. Cummings . 2007. “Frontal‐Subcortical Circuitry and Behavior.” Dialogues in Clinical Neuroscience 9, no. 2: 141–151. 17726913 10.31887/DCNS.2007.9.2/rbonelli PMC3181854

[hbm70364-bib-0012] Bostan, A. C. , R. P. Dum , and P. L. Strick . 2010. “The Basal Ganglia Communicate With the Cerebellum.” Proceedings of the National Academy of Sciences 107, no. 18: 8452–8456. 10.1073/pnas.1000496107 PMC2889518 20404184

[hbm70364-bib-0013] Bruining, H. , R. Hardstone , E. L. Juarez‐Martinez , et al. 2020. “Measurement of Excitation‐Inhibition Ratio in Autism Spectrum Disorder Using Critical Brain Dynamics.” Scientific Reports 10, no. 1: 9195. 32513931 10.1038/s41598-020-65500-4 PMC7280527

[hbm70364-bib-0014] Calhoun, V. D. , and T. Adali . 2012. “Multisubject Independent Component Analysis of fMRI: A Decade of Intrinsic Networks, Default Mode, and Neurodiagnostic Discovery.” IEEE Reviews in Biomedical Engineering 5: 60–73. 23231989 10.1109/RBME.2012.2211076 PMC4433055

[hbm70364-bib-0015] Calhoun, V. D. , J. Liu , and T. Adalı . 2009. “A Review of Group Ica for fMRI Data and Ica for Joint Inference of Imaging, Genetic, and Erp Data.” NeuroImage 45, no. 1: S163–S172. 19059344 10.1016/j.neuroimage.2008.10.057 PMC2651152

[hbm70364-bib-0016] Calhoun, V. D. , R. F. Silva , T. Adalı , and S. Rachakonda . 2015. “Comparison of Pca Approaches for Very Large Group Ica.” NeuroImage 118: 662–666. 26021216 10.1016/j.neuroimage.2015.05.047 PMC4554805

[hbm70364-bib-0017] Charatan, D. , S. L. Li , A. Tagliasacchi , and V. Sitzmann . 2024. “Pixelsplat: 3d Gaussian Splats From Image Pairs for Scalable Generalizable 3d Reconstruction.” In Proceedings of the IEEE/CVF Conference on Computer Vision and Pattern Recognition, 19457–19467. IEEE.

[hbm70364-bib-0018] Cui, Y. , Z. Liu , Y. Chen , et al. 2024. “Retrieval‐Augmented Multiple Instance Learning.” Advances in Neural Information Processing Systems 36: 24859–24878.

[hbm70364-bib-0019] Das, T. K. , J. Kumar , S. Francis , P. F. Liddle , and L. Palaniyappan . 2020. “Parietal Lobe and Disorganisation Syndrome in Schizophrenia and Psychotic Bipolar Disorder: A Bimodal Connectivity Study.” Psychiatry Research: Neuroimaging 303: 111139. 32707490 10.1016/j.pscychresns.2020.111139

[hbm70364-bib-0020] de Geus, D. , and G. Dubbelman . 2024. “Task‐Aligned Part‐Aware Panoptic Segmentation Through Joint Object‐Part Representations.” In Proceedings of the IEEE/CVF Conference on Computer Vision and Pattern Recognition, 3174–3183. IEEE.

[hbm70364-bib-0021] Ding, S.‐L. , J. J. Royall , S. M. Sunkin , et al. 2016. “Comprehensive Cellular‐Resolution Atlas of the Adult Human Brain.” Journal of Comparative Neurology 524, no. 16: 3127–3481. 27418273 10.1002/cne.24080 PMC5054943

[hbm70364-bib-0022] Doucet, G. E. , M. J. Luber , P. Balchandani , I. E. Sommer , and S. Frangou . 2019. “Abnormal Auditory Tonotopy in Patients With Schizophrenia.” npj Schizophrenia 5, no. 1: 1–6. 31578332 10.1038/s41537-019-0084-x PMC6775081

[hbm70364-bib-0023] Du, Y. , Z. Fu , J. Sui , et al. 2020. “Neuromark: An Automated and Adaptive Ica Based Pipeline to Identify Reproducible fMRI Markers of Brain Disorders.” NeuroImage: Clinical 28: 102375. 32961402 10.1016/j.nicl.2020.102375 PMC7509081

[hbm70364-bib-0024] Eickhoff, S. B. , B. Thirion , G. Varoquaux , and D. Bzdok . 2015. “Connectivity‐Based Parcellation: Critique and Implications.” Human Brain Mapping 36, no. 12: 4771–4792. 26409749 10.1002/hbm.22933 PMC6869530

[hbm70364-bib-0025] Faghiri, A. , A. Iraji , E. Damaraju , J. Turner , and V. D. Calhoun . 2021. “A Unified Approach for Characterizing Static/Dynamic Connectivity Frequency Profiles Using Filter Banks.” Network Neuroscience 5, no. 1: 56–82. 33688606 10.1162/netn_a_00155 PMC7935048

[hbm70364-bib-0026] Fan, L. , H. Li , J. Zhuo , et al. 2016. “The Human Brainnetome Atlas: A New Brain Atlas Based on Connectional Architecture.” Cerebral Cortex 26, no. 8: 3508–3526. 27230218 10.1093/cercor/bhw157 PMC4961028

[hbm70364-bib-0027] Forlim, C. G. , L. Klock , J. Bächle , et al. 2020. “Reduced Resting‐State Connectivity in the Precuneus Is Correlated With Apathy in Patients With Schizophrenia.” Scientific Reports 10, no. 1: 2616. 32054907 10.1038/s41598-020-59393-6 PMC7018974

[hbm70364-bib-0028] Fransson, P. , and G. Marrelec . 2008. “The Precuneus/Posterior Cingulate Cortex Plays a Pivotal Role in the Default Mode Network: Evidence From a Partial Correlation Network Analysis.” NeuroImage 42, no. 3: 1178–1184. 18598773 10.1016/j.neuroimage.2008.05.059

[hbm70364-bib-0029] Fransson, P. , and M. Strindberg . 2023. “Brain Network Integration, Segregation and Quasi‐Periodic Activation and Deactivation During Tasks and Rest.” NeuroImage 268: 119890. 36681135 10.1016/j.neuroimage.2023.119890

[hbm70364-bib-0030] Gong, X. , N. Bisht , and G. Xu . 2024. “Does Label Smoothing Help Deep Partial Label Learning?” In Forty‐First International Conference on Machine Learning. Curran Associates, Inc.

[hbm70364-bib-0031] Grafton, S. T. , and L. J. Volz . 2019. “From Ideas to Action: The Prefrontal–Premotor Connections That Shape Motor Behavior.” In Handbook of Clinical Neurology, vol. 163, 237–255. Elsevier. 31590733 10.1016/B978-0-12-804281-6.00013-6

[hbm70364-bib-0032] Haggerty, D. C. , and D. Ji . 2015. “Activities of Visual Cortical and Hippocampal Neurons Co‐Fluctuate in Freely Moving Rats During Spatial Behavior.” eLife 4: e08902. 26349031 10.7554/eLife.08902 PMC4595967

[hbm70364-bib-0033] Han, B. , Q. Yao , T. Liu , et al. 2020. “A Survey of Label‐Noise Representation Learning: Past, Present and Future,” arXiv preprint arXiv:2011.04406.

[hbm70364-bib-0034] Hawrylycz, M. J. , E. S. Lein , A. L. Guillozet‐Bongaarts , et al. 2012. “An Anatomically Comprehensive Atlas of the Adult Human Brain Transcriptome.” Nature 489, no. 7416: 391–399. 22996553 10.1038/nature11405 PMC4243026

[hbm70364-bib-0035] Hindriks, R. , M. H. Adhikari , Y. Murayama , et al. 2016. “Can Sliding‐Window Correlations Reveal Dynamic Functional Connectivity in Resting‐State fMRI?” NeuroImage 127: 242–256. 26631813 10.1016/j.neuroimage.2015.11.055 PMC4758830

[hbm70364-bib-0036] Honari, H. , A. S. Choe , and M. A. Lindquist . 2021. “Evaluating Phase Synchronization Methods in fMRI: A Comparison Study and New Approaches.” NeuroImage 228: 117704. 33385554 10.1016/j.neuroimage.2020.117704 PMC8011682

[hbm70364-bib-0037] Honari, H. , and M. A. Lindquist . 2022. “Mode Decomposition‐Based Time‐Varying Phase Synchronization for fMRI.” NeuroImage 261: 119519. 35905810 10.1016/j.neuroimage.2022.119519 PMC9451171

[hbm70364-bib-0038] Iraji, A. , J. Chen , N. Lewis , et al. 2024. “Spatial Dynamic Subspaces Encode Sex‐Specific Schizophrenia Disruptions in Transient Network Overlap and Their Links to Genetic Risk.” Biological Psychiatry 96, no. 3: 188–197. 38070846 10.1016/j.biopsych.2023.12.002 PMC11156799

[hbm70364-bib-0039] Iraji, A. , T. P. Deramus , N. Lewis , et al. 2019. “The Spatial Chronnectome Reveals a Dynamic Interplay Between Functional Segregation and Integration.” Human Brain Mapping 40, no. 10: 3058–3077. 30884018 10.1002/hbm.24580 PMC6548674

[hbm70364-bib-0040] Iraji, A. , A. Faghiri , Z. Fu , et al. 2022. “Moving Beyond the ‘Cap'of the Iceberg: Intrinsic Connectivity Networks in fMRI Are Continuously Engaging and Overlapping.” NeuroImage 251: 119013. 35189361 10.1016/j.neuroimage.2022.119013 PMC9107614

[hbm70364-bib-0041] Iraji, A. , Z. Fu , E. Damaraju , et al. 2019. “Spatial Dynamics Within and Between Brain Functional Domains: A Hierarchical Approach to Study Time‐Varying Brain Function.” Human Brain Mapping 40, no. 6: 1969–1986. 30588687 10.1002/hbm.24505 PMC6692083

[hbm70364-bib-0042] Iraji, A. , R. Miller , T. Adali , and V. D. Calhoun . 2020. “Space: A Missing Piece of the Dynamic Puzzle.” Trends in Cognitive Sciences 24, no. 2: 135–149. 31983607 10.1016/j.tics.2019.12.004 PMC7809367

[hbm70364-bib-0043] Jobson, D. D. , Y. Hase , A. N. Clarkson , and R. N. Kalaria . 2021. “The Role of the Medial Prefrontal Cortex in Cognition, Ageing and Dementia.” Brain Communications 3, no. 3: fcab125. 34222873 10.1093/braincomms/fcab125 PMC8249104

[hbm70364-bib-0044] Kalluri, T. , W. Wang , H. Wang , M. Chandraker , L. Torresani , and D. Tran . 2024. “Open‐World Instance Segmentation: Top‐Down Learning With Bottom‐Up Supervision.” In Proceedings of the IEEE/CVF Conference on Computer Vision and Pattern Recognition, 2693–2703. IEEE.

[hbm70364-bib-0045] Karniol‐Tambour, O. , D. M. Zoltowski , E. M. Diamanti , et al. 2024. “Modeling State‐Dependent Communication Between Brain Regions with Switching Nonlinear Dynamical Systems,” in The Twelfth International Conference on Learning Representations.

[hbm70364-bib-0046] Kazemivash, B. , and V. D. Calhoun . 2020. “Bparc: A Novel Spatio‐Temporal (4d) Data‐Driven Brain Parcellation Scheme Based on Deep Residual Networks.” In 2020 IEEE 20th International Conference on Bioinformatics and Bioengineering (BIBE), 1071–1076. IEEE.

[hbm70364-bib-0047] Kazemivash, B. , and V. D. Calhoun . 2022a. “A Novel 5d Brain Parcellation Approach Based on Spatio‐Temporal Encoding of Resting fMRI Data From Deep Residual Learning.” Journal of Neuroscience Methods 369: 109478. 35031344 10.1016/j.jneumeth.2022.109478 PMC9394484

[hbm70364-bib-0048] Kazemivash, B. , and V. D. Calhoun . 2022b. “A 5d Approach to Study Spatio‐Temporal Dynamism of Resting‐State Brain Networks in Schizophrenia.” In 2022 44th Annual International Conference of the IEEE Engineering in Medicine & Biology Society (EMBC), 3737–3740. IEEE. 10.1109/EMBC48229.2022.9871362 36085717

[hbm70364-bib-0049] Kazemivash, B. , T. G. van Erp , P. Kochunov , and V. D. Calhoun . 2023. “A Deep Residual Model for Characterization of 5d Spatiotemporal Network Dynamics Reveals Widespread Spatiodynamic Changes in Schizophrenia.” Frontiers in Neuroimaging 2: 1097523. 37554628 10.3389/fnimg.2023.1097523 PMC10406273

[hbm70364-bib-0050] Ke, B. , A. Obukhov , S. Huang , N. Metzger , R. C. Daudt , and K. Schindler . 2024. “Repurposing Diffusion‐Based Image Generators for Monocular Depth Estimation.” In Proceedings of the IEEE/CVF Conference on Computer Vision and Pattern Recognition, 9492–9502. IEEE.

[hbm70364-bib-0051] Kim, D.‐J. , A. B. Moussa‐Tooks , A. R. Bolbecker , et al. 2020. “Cerebellar–Cortical Dysconnectivity in Resting‐State Associated With Sensorimotor Tasks in Schizophrenia.” Human Brain Mapping 41, no. 11: 3119–3132. 32250008 10.1002/hbm.25002 PMC7336143

[hbm70364-bib-0052] Klein, J. C. , M. F. Rushworth , T. E. Behrens , et al. 2010. “Topography of Connections Between Human Prefrontal Cortex and Mediodorsal Thalamus Studied With Diffusion Tractography.” NeuroImage 51, no. 2: 555–564. 20206702 10.1016/j.neuroimage.2010.02.062 PMC2877805

[hbm70364-bib-0053] Lawrence, R. M. , E. W. Bridgeford , P. E. Myers , et al. 2021. “Standardizing Human Brain Parcellations.” Scientific Data 8, no. 1: 78. 33686079 10.1038/s41597-021-00849-3 PMC7940391

[hbm70364-bib-0054] Leech, R. , and D. J. Sharp . 2014. “The Role of the Posterior Cingulate Cortex in Cognition and Disease.” Brain 137, no. 1: 12–32. 23869106 10.1093/brain/awt162 PMC3891440

[hbm70364-bib-0055] Li, M. , L. Dahmani , D. Wang , et al. 2021. “Co‐Activation Patterns Across Multiple Tasks Reveal Robust Anti‐Correlated Functional Networks.” NeuroImage 227: 117680. 33359345 10.1016/j.neuroimage.2020.117680 PMC8034806

[hbm70364-bib-0056] Li, W. , C. Li , Y. Wang , and A. Wu . 2024. “Multi‐region markovian gaussian process: An efficient method to discover directional communications across multiple brain regions,” arXiv preprint arXiv:2402.02686. PMC11526605 39483393

[hbm70364-bib-0057] Li, Z. , X. Wang , X. Liu , and J. Jiang . 2024. “Binsformer: Revisiting Adaptive Bins for Monocular Depth Estimation.” IEEE Transactions on Image Processing 33: 3964–3976. 38913511 10.1109/TIP.2024.3416065

[hbm70364-bib-0058] Liégeois, R. , J. Li , R. Kong , et al. 2019. “Resting Brain Dynamics at Different Timescales Capture Distinct Aspects of Human Behavior.” Nature Communications 10, no. 1: 2317. 10.1038/s41467-019-10317-7 PMC6534566 31127095

[hbm70364-bib-0059] Lin, Q.‐H. , J. Liu , Y.‐R. Zheng , H. Liang , and V. D. Calhoun . 2010. “Semiblind Spatial ICA of fMRI Using Spatial Constraints.” Human Brain Mapping 31, no. 7: 1076–1088. 20017117 10.1002/hbm.20919 PMC2891131

[hbm70364-bib-0060] Lin, X. , F. Petroni , G. Bertasius , M. Rohrbach , S.‐F. Chang , and L. Torresani . 2022. “Learning to Recognize Procedural Activities With Distant Supervision.” In Proceedings of the IEEE/CVF Conference on Computer Vision and Pattern Recognition, 13853–13863. IEEE.

[hbm70364-bib-0061] Liu, B. , I. Ben Ayed , A. Galdran , and J. Dolz . 2022. “The Devil Is in the Margin: Margin‐Based Label Smoothing for Network Calibration.” In Proceedings of the IEEE/CVF Conference on Computer Vision and Pattern Recognition, 80–88. IEEE.

[hbm70364-bib-0062] Liu, X. , N. Zhang , C. Chang , and J. H. Duyn . 2018. “Co‐Activation Patterns in Resting‐State fmri Signals.” NeuroImage 180: 485–494. 29355767 10.1016/j.neuroimage.2018.01.041 PMC6082734

[hbm70364-bib-0063] Lu, Y. , Y. Zhang , B. Han , Y.‐m. Cheung , and H. Wang . 2023. “Label‐Noise Learning With Intrinsically Long‐Tailed Data.” In Proceedings of the IEEE/CVF International Conference on Computer Vision, 1369–1378. IEEE.

[hbm70364-bib-0064] Luo, A. , X. Li , F. Yang , J. Liu , H. Fan , and S. Liu . 2024. “Flowdiffuser: Advancing Optical Flow Estimation With Diffusion Models.” In Proceedings of the IEEE/CVF Conference on Computer Vision and Pattern Recognition, 19167–19176. IEEE.

[hbm70364-bib-0065] Luo, X. , A. Luo , Z. Wang , C. Lin , B. Zeng , and S. Liu . 2024. “Efficient Meshflow and Optical Flow Estimation From Event Cameras.” In Proceedings of the IEEE/CVF Conference on Computer Vision and Pattern Recognition, 19198–19207. IEEE.

[hbm70364-bib-0066] Lv, H. , Z. Yue , Q. Sun , B. Luo , Z. Cui , and H. Zhang . 2023. “Unbiased Multiple Instance Learning for Weakly Supervised Video Anomaly Detection.” In Proceedings of the IEEE/CVF Conference on Computer Vision and Pattern Recognition, 8022–8031. IEEE.

[hbm70364-bib-0067] Marino, M. , and D. Mantini . 2024. “Human Brain Imaging With High‐Density Electroencephalography: Techniques and Applications.” Journal of Physiology, Online ahead of print, August 22. 10.1113/JP286639. PMC12810243 39173191

[hbm70364-bib-0068] Matsui, T. , T. Q. Pham , K. Jimura , and J. Chikazoe . 2022. “On Co‐Activation Pattern Analysis and Non‐Stationarity of Resting Brain Activity.” NeuroImage 249: 118904. 35031473 10.1016/j.neuroimage.2022.118904

[hbm70364-bib-0069] Mayer, A. R. , D. Ruhl , F. Merideth , et al. 2013. “Functional Imaging of the Hemodynamic Sensory Gating Response in Schizophrenia.” Human Brain Mapping 34, no. 9: 2302–2312. 22461278 10.1002/hbm.22065 PMC4020570

[hbm70364-bib-0070] Moghimi, P. , A. T. Dang , Q. Do , T. I. Netoff , K. O. Lim , and G. Atluri . 2022. “Evaluation of Functional Mri‐Based Human Brain Parcellation: A Review.” Journal of Neurophysiology 128, no. 1: 197–217. 35675446 10.1152/jn.00411.2021 PMC9291407

[hbm70364-bib-0071] Müller, R. , S. Kornblith , and G. E. Hinton . 2019. “When Does Label Smoothing Help?” Advances in Neural Information Processing Systems 32: 4696–4705.

[hbm70364-bib-0072] Murphy, K. P. 2022. Probabilistic Machine Learning: An Introduction. MIT Press.

[hbm70364-bib-0073] Nguyen, H. C. , T. Wang , J. M. Alvarez , and M. Liu . 2024. “Mining Supervision for Dynamic Regions in Self‐Supervised Monocular Depth Estimation.” In Proceedings of the IEEE/CVF Conference on Computer Vision and Pattern Recognition, 10446–10455. IEEE.

[hbm70364-bib-0074] Omidvarnia, A. , M. Pedersen , J. M. Walz , D. N. Vaughan , D. F. Abbott , and G. D. Jackson . 2016. “Dynamic Regional Phase Synchrony (Dreps) an Instantaneous Measure of Local fMRI Connectivity Within Spatially Clustered Brain Areas.” Human Brain Mapping 37, no. 5: 1970–1985. 27019380 10.1002/hbm.23151 PMC6867553

[hbm70364-bib-0075] O'reilly, J. X. , C. F. Beckmann , V. Tomassini , N. Ramnani , and H. Johansen‐Berg . 2010. “Distinct and Overlapping Functional Zones in the Cerebellum Defined by Resting State Functional Connectivity.” Cerebral Cortex 20, no. 4: 953–965. 19684249 10.1093/cercor/bhp157 PMC2837094

[hbm70364-bib-0076] Pergola, G. , P. Selvaggi , S. Trizio , A. Bertolino , and G. Blasi . 2015. “The Role of the Thalamus in Schizophrenia From a Neuroimaging Perspective.” Neuroscience & Biobehavioral Reviews 54: 57–75. 25616183 10.1016/j.neubiorev.2015.01.013

[hbm70364-bib-0077] Pomarol‐Clotet, E. , E. J. Canales‐Rodríguez , R. Salvador , et al. 2010. “Medial Prefrontal Cortex Pathology in Schizophrenia as Revealed by Convergent Findings From Multimodal Imaging.” Molecular Psychiatry 15, no. 8: 823–830. 20065955 10.1038/mp.2009.146 PMC2927029

[hbm70364-bib-0078] Potkin, S. G. , and J. M. Ford . 2009. “Widespread Cortical Dysfunction in Schizophrenia: The Fbirn Imaging Consortium.” Schizophrenia Bulletin 35, no. 1: 15–18. 19023124 10.1093/schbul/sbn159 PMC2643955

[hbm70364-bib-0079] Qi, Y. , W. Zhao , and X. Wu . 2024. “Relational Distant Supervision for Image Captioning Without Image‐Text Pairs.” Proceedings of the AAAI Conference on Artificial Intelligence 38, no. 5: 4524–4532.

[hbm70364-bib-0080] Ragland, J. , E. Layher , D. Hannula , et al. 2017. “Impact of Schizophrenia on Anterior and Posterior Hippocampus During Memory for Complex Scenes.” NeuroImage: Clinical 13: 82–88. 27942450 10.1016/j.nicl.2016.11.017 PMC5133646

[hbm70364-bib-0081] Raut, R. V. , A. Z. Snyder , and M. E. Raichle . 2020. “Hierarchical Dynamics as a Macroscopic Organizing Principle of the Human Brain.” Proceedings of the National Academy of Sciences of the United States of America 117, no. 34: 20890–20897. 32817467 10.1073/pnas.2003383117 PMC7456098

[hbm70364-bib-0082] Rokham, H. , H. Falakshahi , and V. D. Calhoun . 2023. “A Deep Learning Approach for Psychosis Spectrum Label Noise Detection From Multimodal Neuroimaging Data.” In 2023 45th Annual International Conference of the IEEE Engineering in Medicine & Biology Society (EMBC), 1–4. IEEE. 10.1109/EMBC40787.2023.10339949 38082903

[hbm70364-bib-0083] Rokham, H. , G. Pearlson , A. Abrol , H. Falakshahi , S. Plis , and V. D. Calhoun . 2020. “Addressing Inaccurate Nosology in Mental Health: A Multilabel Data Cleansing Approach for Detecting Label Noise From Structural Magnetic Resonance Imaging Data in Mood and Psychosis Disorders.” Biological Psychiatry: Cognitive Neuroscience and Neuroimaging 5, no. 8: 819–832. 32771180 10.1016/j.bpsc.2020.05.008 PMC7760893

[hbm70364-bib-0084] Ruchalski, K. , and G. M. Hathout . 2012. “A Medley of Midbrain Maladies: A Brief Review of Midbrain Anatomy and Syndromology for Radiologists.” Radiology Research and Practice 2012, no. 1: 258524. 22693668 10.1155/2012/258524 PMC3366251

[hbm70364-bib-0085] Saalmann, Y. B. , and S. Kastner . 2011. “Cognitive and Perceptual Functions of the Visual Thalamus.” Neuron 71, no. 2: 209–223. 21791281 10.1016/j.neuron.2011.06.027 PMC3148184

[hbm70364-bib-0086] Sadaghiani, S. , R. Scheeringa , K. Lehongre , et al. 2012. “Alpha‐Band Phase Synchrony Is Related to Activity in the Fronto‐Parietal Adaptive Control Network.” Journal of Neuroscience 32, no. 41: 14305–14310. 23055501 10.1523/JNEUROSCI.1358-12.2012 PMC4057938

[hbm70364-bib-0087] Savva, A. D. , M. Kassinopoulos , N. Smyrnis , G. K. Matsopoulos , and G. D. Mitsis . 2020. “Effects of Motion Related Outliers in Dynamic Functional Connectivity Using the Sliding Window Method.” Journal of Neuroscience Methods 330: 108519. 31730872 10.1016/j.jneumeth.2019.108519

[hbm70364-bib-0088] Savva, A. D. , G. D. Mitsis , and G. K. Matsopoulos . 2019. “Assessment of Dynamic Functional Connectivity in Resting‐State fMRI Using the Sliding Window Technique.” Brain and Behavior: A Cognitive Neuroscience Perspective 9, no. 4: e01255. 10.1002/brb3.1255 PMC6456784 30884215

[hbm70364-bib-0089] Seeburger, D. T. , N. Xu , M. Ma , et al. 2024. “Time‐Varying Functional Connectivity Predicts Fluctuations in Sustained Attention in a Serial Tapping Task.” Cognitive, Affective, & Behavioral Neuroscience 24, no. 1: 111–125. 10.3758/s13415-024-01156-1 PMC10979291 38253775

[hbm70364-bib-0090] Selemon, L. , and N. Zecevic . 2015. “Schizophrenia: A Tale of Two Critical Periods for Prefrontal Cortical Development.” Translational Psychiatry 5, no. 8: e623. 26285133 10.1038/tp.2015.115 PMC4564568

[hbm70364-bib-0091] Shakil, S. , C.‐H. Lee , and S. D. Keilholz . 2016. “Evaluation of Sliding Window Correlation Performance for Characterizing Dynamic Functional Connectivity and Brain States.” NeuroImage 133: 111–128. 26952197 10.1016/j.neuroimage.2016.02.074 PMC4889509

[hbm70364-bib-0092] Soler‐Vidal, J. , P. Fuentes‐Claramonte , P. Salgado‐Pineda , et al. 2022. “Brain Correlates of Speech Perception in Schizophrenia Patients With and Without Auditory Hallucinations.” PLoS One 17, no. 12: e0276975. 36525414 10.1371/journal.pone.0276975 PMC9757556

[hbm70364-bib-0093] Sporns, O. 2022. “The Complex Brain: Connectivity, Dynamics, Information.” Trends in Cognitive Sciences 26, no. 12: 1066–1067. 36207260 10.1016/j.tics.2022.08.002

[hbm70364-bib-0094] Stevens, F. L. , R. A. Hurley , and K. H. Taber . 2011. “Anterior Cingulate Cortex: Unique Role in Cognition and Emotion.” Journal of Neuropsychiatry and Clinical Neurosciences 23, no. 2: 121–125. 21677237 10.1176/jnp.23.2.jnp121

[hbm70364-bib-0095] Sun, S. , W. Wang , A. Howard , Q. Yu , P. Torr , and L.‐C. Chen . 2024. “Remax: Relaxing for Better Training on Efficient Panoptic Segmentation.” Advances in Neural Information Processing Systems 36: 73480–73496.

[hbm70364-bib-0096] Sung, C. , W. Kim , J. An , W. Lee , H. Lim , and H. Myung . 2024. “Contextrast: Contextual Contrastive Learning for Semantic Segmentation.” In Proceedings of the IEEE/CVF Conference on Computer Vision and Pattern Recognition, 3732–3742. IEEE.

[hbm70364-bib-0097] Szymanowicz, S. , C. Rupprecht , and A. Vedaldi . 2024. “Splatter Image: Ultra‐Fast Single‐View 3d Reconstruction.” In Proceedings of the IEEE/CVF Conference on Computer Vision and Pattern Recognition, 10208–10217. IEEE.

[hbm70364-bib-0098] Thompson, G. J. , W.‐J. Pan , M. E. Magnuson , D. Jaeger , and S. D. Keilholz . 2014. “Quasi‐Periodic Patterns (Qpp): Large‐Scale Dynamics in Resting State fMRI That Correlate With Local Infraslow Electrical Activity.” NeuroImage 84: 1018–1031. 24071524 10.1016/j.neuroimage.2013.09.029 PMC3869452

[hbm70364-bib-0099] Turk‐Browne, N. B. 2019. “The Hippocampus as a Visual Area Organized by Space and Time: A Spatiotemporal Similarity Hypothesis.” Vision Research 165: 123–130. 31734633 10.1016/j.visres.2019.10.007 PMC6881556

[hbm70364-bib-0100] Uddin, L. Q. , K. Supekar , H. Amin , et al. 2010. “Dissociable Connectivity Within Human Angular Gyrus and Intraparietal Sulcus: Evidence From Functional and Structural Connectivity.” Cerebral Cortex 20, no. 11: 2636–2646. 20154013 10.1093/cercor/bhq011 PMC2951845

[hbm70364-bib-0101] Usrey, W. M. , and H. J. Alitto . 2015. “Visual Functions of the Thalamus.” Annual Review of Vision Science 1, no. 1: 351–371. 10.1146/annurev-vision-082114-035920 PMC5310631 28217740

[hbm70364-bib-0102] van der Stelt, O. , J. A. Lieberman , and A. Belger . 2006. “Attentional Modulation of Early‐Stage Visual Processing in Schizophrenia.” Brain Research 1125, no. 1: 194–198. 17087921 10.1016/j.brainres.2006.09.099 PMC1933501

[hbm70364-bib-0103] Vergara, V. M. , A. Abrol , and V. D. Calhoun . 2019. “An Average Sliding Window Correlation Method for Dynamic Functional Connectivity.” Human Brain Mapping 40, no. 7: 2089–2103. 30659699 10.1002/hbm.24509 PMC6865616

[hbm70364-bib-0104] Walton, E. , D. P. Hibar , T. G. van Erp , et al. 2017. “Left Medial Orbitofrontal Cortical Thinning Is Associated With Negative Symptom Severity in Schizophrenia: A Meta‐Analysis by the Enigma‐Schizophrenia Consortium.” Psychological Medicine 48, no. 1: 82. 28545597 10.1017/S0033291717001283 PMC5826665

[hbm70364-bib-0105] Wang, H. , L.‐L. Zeng , Y. Chen , H. Yin , Q. Tan , and D. Hu . 2015. “Evidence of a Dissociation Pattern in Default Mode Subnetwork Functional Connectivity in Schizophrenia.” Scientific Reports 5, no. 1: 14655. 26419213 10.1038/srep14655 PMC4588504

[hbm70364-bib-0106] Wei, Z. , P. Chen , X. Yu , G. Li , J. Jiao , and Z. Han . 2024. “Semantic‐Aware Sam for Point‐Prompted Instance Segmentation.” In Proceedings of the IEEE/CVF Conference on Computer Vision and Pattern Recognition, 3585–3594. IEEE.

[hbm70364-bib-0107] Weng, Y. , M. Han , H. He , et al. 2024. “Mask Propagation for Efficient Video Semantic Segmentation.” Advances in Neural Information Processing Systems 36: 7170–7183.

[hbm70364-bib-0108] Wible, C. G. , A. P. Preus , and R. Hashimoto . 2009. “A Cognitive Neuroscience View of Schizophrenic Symptoms: Abnormal Activation of a System for Social Perception and Communication.” Brain Imaging and Behavior 3: 85–110. 19809534 10.1007/s11682-008-9052-1 PMC2757313

[hbm70364-bib-0109] Woodward, N. D. , B. Rogers , and S. Heckers . 2011. “Functional Resting‐State Networks Are Differentially Affected in Schizophrenia.” Schizophrenia Research 130, no. 1–3: 86–93. 21458238 10.1016/j.schres.2011.03.010 PMC3139756

[hbm70364-bib-0110] Xia, C. , X. Wang , F. Lv , X. Hao , and Y. Shi . 2024. “Vit‐Comer: Vision Transformer With Convolutional Multi‐Scale Feature Interaction for Dense Predictions.” In Proceedings of the IEEE/CVF Conference on Computer Vision and Pattern Recognition, 5493–5502. IEEE.

[hbm70364-bib-0111] Yang, X. , L. Yuan , K. Wilber , et al. 2024. “Polymax: General Dense Prediction With Mask Transformer.” In Proceedings of the IEEE/CVF Winter Conference on Applications of Computer Vision, 1050–1061. IEEE.

[hbm70364-bib-0112] Yang, Y. , P.‐T. Jiang , Q. Hou , H. Zhang , J. Chen , and B. Li . 2024. “Multi‐Task Dense Prediction via Mixture of Low‐Rank Experts.” In Proceedings of the IEEE/CVF Conference on Computer Vision and Pattern Recognition, 27927–27937. IEEE.

[hbm70364-bib-0113] Yao, Y. , A. Zhang , X. Han , et al. 2021. “Visual Distant Supervision for Scene Graph Generation.” In Proceedings of the IEEE/CVF International Conference on Computer Vision, 15816–15826. IEEE.

[hbm70364-bib-0114] Yildiz, M. , S. J. Borgwardt , and G. E. Berger . 2011. “Parietal Lobes in Schizophrenia: Do They Matter?” Schizophrenia Research and Treatment 2011, no. 1: 581686. 22937268 10.1155/2011/581686 PMC3420742

[hbm70364-bib-0115] Yoshida, J. , M. Oñate , L. Khatami , J. Vera , F. Nadim , and K. Khodakhah . 2022. “Cerebellar Contributions to the Basal Ganglia Influence Motor Coordination, Reward Processing, and Movement Vigor.” Journal of Neuroscience 42, no. 45: 8406–8415. 36351826 10.1523/JNEUROSCI.1535-22.2022 PMC9665921

[hbm70364-bib-0116] Yuan, T. , F. Wan , M. Fu , et al. 2021. “Multiple Instance Active Learning for Object Detection.” In Proceedings of the IEEE/CVF Conference on Computer Vision and Pattern Recognition, 5330–5339. IEEE.

[hbm70364-bib-0117] Zarghami, T. S. , G.‐A. Hossein‐Zadeh , and F. Bahrami . 2020. “Deep Temporal Organization of fMRI Phase Synchrony Modes Promotes Large‐Scale Disconnection in Schizophrenia.” Frontiers in Neuroscience 14: 214. 32292324 10.3389/fnins.2020.00214 PMC7118690

[hbm70364-bib-0118] Zhang, L. , X. Wang , and H. Liu . 2024. “Functional Magnetic Resonance Imaging: An Overview of Technical Advances and Clinical Applications.” In AI‐Driven Innovations in Digital Healthcare: Emerging Trends, Challenges, and Applications, 120–140. Medical Information Science Reference.

[hbm70364-bib-0120] Zhang, Y. , L. Lin , D. Zhou , et al. 2024. “Age‐Related Unstable Transient States and Imbalanced Activation Proportion of Brain Networks in People With Autism Spectrum Disorder: A Resting‐State fMRI Study Using Co‐Activation Pattern Analyses.” Network Neuroscience 8: 1173–1191. 39735491 10.1162/netn_a_00396 PMC11674577

[hbm70364-bib-0121] Zhao, Y. , T. Kirschenhofer , M. Harvey , and G. Rainer . 2024. “Mediodorsal Thalamus and Ventral Pallidum Contribute to Subcortical Regulation of the Default Mode Network.” Communications Biology 7, no. 1: 891. 39039239 10.1038/s42003-024-06531-9 PMC11263694

